# Prostaglandin I_2_ Attenuates Prostaglandin E_2_-Stimulated Expression of Interferon γ in a β-Amyloid Protein- and NF-κB-Dependent Mechanism

**DOI:** 10.1038/srep20879

**Published:** 2016-02-12

**Authors:** Pu Wang, Pei-Pei Guan, Xin Yu, Li-Chao Zhang, Ya-Nan Su, Zhan-You Wang

**Affiliations:** 1 College of Life and Health Sciences, Northeastern University, Shenyang 110819, P. R. China

## Abstract

Cyclooxygenase-2 (COX-2) has been recently identified as being involved in the pathogenesis of Alzheimer’s disease (AD). However, the role of an important COX-2 metabolic product, prostaglandin (PG) I_2_, in AD development remains unknown. Using mouse-derived astrocytes as well as APP/PS1 transgenic mice as model systems, we firstly elucidated the mechanisms of interferon γ (IFNγ) regulation by PGE_2_ and PGI_2_. Specifically, PGE_2_ accumulation in astrocytes activated the ERK1/2 and NF-κB signaling pathways by phosphorylation, which resulted in IFNγ expression. In contrast, the administration of PGI_2_ attenuated the effects of PGE_2_ on stimulating the production of IFNγ *via* inhibiting the translocation of NF-κB from the cytosol to the nucleus. Due to these observations, we further studied these prostaglandins and found that both PGE_2_ and PGI_2_ increased Aβ_1–42_ levels. In detail, PGE_2_ induced IFNγ expression in an Aβ_1–42_-dependent manner, whereas PGI_2_-induced Aβ_1–42_ production did not alleviate cells from IFNγ inhibition by PGI_2_ treatment. More importantly, our data also revealed that not only Aβ_1–42_ oligomer but also fibrillar have the ability to induce the expression of IFNγ via stimulation of NF-κB nuclear translocation in astrocytes of APP/PS1 mice. The production of IFNγ finally accelerated the deposition of Aβ_1–42_ in β-amyloid plaques.

Alzheimer’s diseases (AD) is the most common cause of dementia in aged people and is characterized clinically by cognitive decline and pathologically by the accumulation of β-amyloid protein (Aβ) and hyperphosphorylation of tau in the brain^1^. It has been generally accepted that neuroinflammation is involved in Aβ deposition and tau phosphorylation, which contribute to the progression of AD^2^,^3^. Although the mechanisms of neuroinflammation in AD have not yet been elucidated, cyclooxygenase-2 (COX-2) has been suggested as having a potential role in neuroinflammation. This is due to its metabolic products, the prostaglandins (PGs), which include PGE_2_, PGD_2_ [and its dehydration end product, 15-deoxy-Δ^4^,^5^-PGJ_2_ (15d-PGJ_2_)], PGI_2_, PGF_2α_ and TXA_2_^4^. Among these PGs, both PGE_2_ and PGI_2_ are potential mediators of inflammation^5^,^6^. For example, PGE_2_ is involved in all processes leading to the classic signs of inflammation: redness, swelling and pain^9^. Pain results from the action of PGE_2_ on peripheral sensory neurons and on central sites within the spinal cord and the brain^7^. Apart from PGE_2_, PGI_2_ signaling facilitated joint inflammation in a mouse model of collagen-induced arthritis, while the administration of a PGI_2_ antagonist reduced pain and inflammation in rodent models of hyperalgesia and chronic arthritis^8^. In contrast to the seemingly pro-inflammatory properties of PGI_2_, there is still debate about its effects in certain conditions^2^. For example, PGI_2_ has been studied as a potentially important suppressor of allergen-induced inflammation^2^. Thus, the effects of PGI_2_ on inflammatory reactions of peripheral tissues are still uncertain rather than neuroinflammation.

Although we could not find direct evidence that demonstrates the relationship between PGs and neuroinflammation, a growing body of research reveals that both PGE_2_ and PGI_2_ has the ability to regulate the synthesis of cytokines^9^. For example, our prior works demonstrated that PGE_2_ has ability to stimulate the expression of IL-1β in A172 cells^10^. In addition, TNF-α was also stimulated in PGE_2_-stimulated SH-SY5Y cells^11^. In astrocytes, PGE_2_ also showed its stimulatory effects on the expression of IL-6^12^ and IFNγ^13^,^14^. Similar to PGE_2_, PGI_2_ analogues including iloprost and treprostinil treatment induced IL-10 expression but suppressed TNF-α expression in human myeloid dendritic cells^9^. Additionally, Wahlstrom *et al.*^15^ reported that when compared to a placebo treatment, the administration of the PGI_2_ analogue epoprostenol significantly decreased C-reactive protein (CRP) and generally decreased IL-6 levels in patients with severe traumatic brain injury. Following from this observation, Schuh *et al.*^16^ reported that the early induction of PGI_2_ at the site of traumatic injury resulted in the aggregation of IL-1β-expressing macrophages as a critical cause of neuropathic pain. Apart from interleukins and TNF-α, Strassheim *et al.*^17^ reported that PGI_2_ inhibits interferon γ (IFNγ)-stimulated cytokine expression in human monocytes. However, the regulatory mechanisms between PGI_2_ and IFNγ, including the role of PGI_2_ in regulating the expression of IFNγ during the course of AD development are often not studied.

Although little is known about the relationship between PGE_2_/I_2_ and IFNγ, IFNγ has already been suggested to regulate the pathogenesis of AD^18^. For example, IFNγ treatment activates the promoter of BACE-1 in human U373MG astrocytoma cells^19^. Additionally, IFNγ stimulates β-secretase expression and sAPPβ production in mouse astrocytes^20^. Yamamoto *et al.*^21^ also found that IFNγ regulates amyloid plaque (AP) deposition in Swedish mutant APP transgenic mice. Apart from β-secretase, it has also been reported that IFNγ production has the ability to accelerate γ-secretase cleavage of APP^22^ by upregulating the expression of presenillin 2 (PS2) in human neuronal cells^23^. When considered together, these data prompted us to investigate the roles of PGE_2_ and PGI_2_ in regulating the expression of IFNγ during the course of AD development.

To understand the relationship between PGs and IFNγ, we first delineated the signaling pathway of IFNγ upregulation in APP/PS1 mice. Specifically, we demonstrated that PGE_2_ induction at the early stage of AD stimulates the expression of IFNγ via Aβ_1–42_-dependent NF-κB-activating pathways. In contrast, PGI_2_ attenuated the effects of PGE_2_ on stimulating the expression of IFNγ by depressing NF-κB nuclear translocation. Although PGI_2_ also has the ability to enhance the production of Aβ_1–42_, Aβ_1–42_ could not alleviate IFNγ inhibition from PGI_2_ treatment. In addition, not only Aβ oligomers but also Aβ fibrils have ability to stimulate the expression of IFNγ, which is responsible for sustaining high levels of IFNγ during the course of AD development. Reciprocally, IFNγ accumulation in or secretion from astrocytes accelerates the Aβ deposition in APs. Therefore, PGE_2_ and PGI_2_ have opposing effects on IFNγ expression, which is responsible for accelerating Aβ_1–42_ deposition in APs during the course of AD development.

## Materials and Methods

### Reagents

PGI_2_, PGE_2_, Aβ_1–42_ and the inhibitors NS398, U0126, and KT5720 were obtained from Sigma-Aldrich Corp (St. Louis, MO, USA). Antibodies against β-actin, ERK1/2, p-ERK1/2 (Thr 202/Tyr 204), NF-κB, p-NF-κB (Ser 536), p-NF-κB (Ser 276), IκB, IFNγ, BACE-1, PS1, PS2, GFAP and human Aβ were purchased from Cell Signaling Technology, Inc. (Danvers, MA, USA). sAPPα and sAPPβ antibodies were obtained from IBL International Corp. (Toronto, ON, Canada). The human IFNγ and IFNγ enzyme immunoassay kits were obtained from Raybiotech, Inc. (Norcross, GA, USA). Human or mouse Aβ_1–42_ ELISA kits were obtained from Invitrogen (Carlsbad, CA, USA). ERK1/2, p65 and scramble siRNA were obtained from Cell Signaling Technology, Inc. (Danvers, MA, USA). The chromatin immunoprecipitation (ChIP) EZ-ChIP kit was purchased from Upstate Biotechnology. All reagents for the qRT-PCR and SDS-PAGE experiments were purchased from Bio-Rad Laboratories. All other reagents were from Invitrogen (Carlsbad, CA, USA) unless otherwise specified.

### Transgenic mice and treatments

The female wild type (WT) or APP/PS1 transgenic mice [B6C3-Tg (APPswe, PSEN1dE9) 85Dbo/J (Stock Number: 004462)] were obtained from The Jackson laboratory (Bar Harbor, ME, USA)^24^. Genotyping was performed at 3–4 weeks after birth. The mice were housed in a controlled environment under a standard room temperature, relative humidity and 12-h light/dark cycle with free access to food and water. Mice were randomly separated into several groups and each group contains 10 mice. Mice at 6 months of age were injected (i.c.v) with PGE_2_ (2 μg/5 μl) or PGI_2_ (2 μg/5 μl) in the absence or presence of Aβ antibody (1 μg/5 μl) or Aβ oligomers (1 μg/5 μl) for 24 h before determining the expression of IFNγ. In select experiments, WT mice at 6 months of age were injected with 5 μl CSF that was collected from APP/PS1 mice at 6 months of age [in the absence or presence of Aβ antibody (1 μg/5 μl)] at 24 h prior to IFNγ gene expression studies. In separate experiments, the WT mice were injected (i.c.v) with Aβ oligomers (1 μg/5 μl) or fibrils (1 μg/5 μl) at 24 h prior to IFNγ gene expression studies. In distinct experiments, IFNγ (10 ng/20 μl/d) was nasally administered to 3-months-old WT or APP/PS1 mice for 7 days, 3 months or 6 months before determining the Aβ deposition in APs. The general health and body weights of animals were monitored every day. The brains of animals from the different groups were collected under anesthesia and perfusion as previously described^25^.

### Aβ_1–42_ preparation

The methods for preparing Aβ oligomers or fibrils had been described previously^26^,^27^,^28^. In brief, freeze-drying Aβ_1–42_ protein (Stock Number: A9810, Sigma, St. Louis, MO, USA) was initially monomerized by dissolving it to a final concentration of 1 μg/μl in 100% hexafluoroisopropanal (HFIP) and the solution was aliquoted in sterile eppendorf tubes. HFIP was then evaporated under vacuum and the peptide was stored at −20 °C before reconstituent. For preparing Aβ_1–42_ oligomers, the peptide was initially resuspended in dimethylsulfoxide (DMSO) to 20 μg/μl with water bath ultrasonication for 10 min and the solution was then diluted to a final concentration of 0.2 mg/ml in phenol red-free F-12 media, and incubated at 4 °C for 24 h. To prepare Aβ_1–42_ fibrils, Aβ_1–42_ was resuspended in sterile Milli Q water and incubated at 37 °C for 1 week before use.

### Intracerebroventricular injection (i.c.v)

NS398, PGE_2_, PGI_2_, Aβ, or anti-human Aβ or vehicle (PBS) solutions were injected (i.c.v) into WT or APP/PS1 transgenic mice as previously described^25^. In selected experiments, the WT mice were injected (i.c.v) with the CSF of APP/PS1 mice. Briefly, stereotaxic injections were placed at the following coordinates from the bregma: mediolateral: −1.0 mm; anteroposterior: −0.22 mm; and dorsoventral: −2.8 mm. Following injections, each mouse recovered spontaneously on a heated pad. The reliability of injection sites was validated by injecting trypan blue dye (Invitrogen) into separate cohorts of mice and observing staining in the cerebral ventricles. Twenty-four hours after injection, mice were harvested under anesthesia and perfusion as previously described^25^.

### Organotypic slice culture of brain tissue

Brain tissues were freshly collected from WT C57BL/6 mice at 6 months of age. Serial sections (400-μm thick) were cut using a chopper without fixation. The tissue sections were immediately cultured in DMEM/high glucose medium with 10% fetal bovine serum (FBS). In a separate set of experiments, the tissues were grown in serum-free medium for an additional 24 h before incubation with Aβ oligomers or fibrils, as previously described^25^. The tissue sections were fixed and immunostained with IFNγ antibody by an immunohistochemical staining kit (Invitrogen, Carlsbad, CA, USA).

### Luciferase assays and live animal imaging

The experiments were performed as previously described^26^. The D1A cells that were transfected with an IFNγ promoter were pre-seeded in one side of a ventricle. PGI_2_, PGE_2 _or vehicle (PBS) solutions were then injected (i.c.v) into the other side of ventricle. At different time intervals, mice were anesthetized and injected (i.c.v) with luciferin into the cerebral ventricle, which was preseeded with D1A cells. The scan was performed exactly after 5 min of luciferin introduction. All images were analyzed using Bruker *in vivo* imaging systems (MS FX PRO, Carestream, U.S.A).

### Cell culture

Mouse astrocyte D1A and neuroblastoma n2a cells were grown (37 °C and 5% CO_2_) on 6-cm tissue culture dishes (10^6 ^cells per dish) in appropriate medium. In a separate set of experiments, the cells were grown in serum-free medium for an additional 24 h before incubation with inhibitors in the absence or presence of PGI_2_ or PGE_2_, as previously described^10^,^25^.

### ChIP Assay

This assay was performed using the EZ ChIP kit following the manufacturer’s instructions (Upstate Biotechnology) as described previously^27^,^28^,^29^,^30^. Forward (F) and reverse (R) primers for IFNγ promoter amplification by qPCR are as follows: F-CGTTGACCCTGAGTGATTTG and R-GTTTCCTTTCGACTCCTTGG.

### Quantitative real-time PCR (qRT-PCR)

qRT-PCR assays were performed with the MiniOpticon Real-Time PCR detection system (Bio-Rad) using total RNA and the GoTaq one-step Real-Time PCR kit with SYBR green (Promega) and the appropriate primers as previously described^31^. The GenBank accession number and forward and reverse primers for mouse GAPDH and BACE-1 are provided in our previous publications^10^,^32^,^33^: mouse IFNγ (NM_008337.3) F-CACGGCACAGTCATTGAAAG, R-ATCAGCAGCGACTCCTTTTC; GFAP (NM_001131020) F-AATGCTGGCTTCAAGGAGAC, R-CTCCAGCGATTCAACCTTTC; PS1 (NM_008943) F-GCTTGTAGGCGCCTTTAGTG, R-CATCTGGGCATTCTGGAAGT; PS2 (NM_011183) F- AAGAACGGGCAGCTCATCTA, R-TCCAGACAGCCAGGAAGAGT. The gene expression values were normalized to those of GAPDH.

### Western blot analysis

Tissues or cells were lysed in radio-immune precipitation assay buffer (25 mM Tris-HCl [pH 7.6], 150 mM NaCl, 1% NP-40, 1% sodium deoxycholate, and 0.1% SDS) that contained a protease inhibitor cocktail (Pierce Chemical Company). The protein content of the cell lysates was determined using the bicinchoninic acid (BCA) protein assay reagent (Pierce Chemical Company). The total protein lysates (4 μg) were separated using SDS-PAGE, transferred to a membrane, and probed with a panel of specific antibodies. In general, primary and secondary antibody was diluted in TBST by the ratio of 1:2000 and 1:5000, respectively. Each membrane was only probed with one antibody. β-actin was used as a loading control. The membrane was visualized by ECL. All western hybridizations were performed at least in triplicate using a different cell preparation each time.

### Immunohistochemistry

Brain tissues were collected from WT or APP/PS1 transgenic mice. In selected experiments, brain tissues were collected after injection (i.c.v) of PGI_2_ (2 μg/5 μl) or PGE_2_ (2 μg/5 μl). Serial sections (5-μm thick) were cut using a paraffin microtome (Leica, RM2235, Germany). Sections were first rehydrated in a graded series of ethanol and submerged in 3% hydrogen peroxide to eliminate endogenous peroxidase activity. The activity of astrocytes was determined by staining GFAP using an immunohistochemical staining kit, following the manufacturer’s instructions (Invitrogen, Carlsbad, CA, USA).

### Immunofluorescence

Brain tissues were collected from WT or APP/PS1 transgenic mice. In selected experiments, brain tissues were collected after injection (i.c.v) of PGI_2_ (2 μg/5 μl) or PGE_2_ (2 μg/5 μl). Serial sections (10-μm thick) were cut using a cryostat (Leica, CM1850, Germany). Slides were stained with IFNγ or Aβ antibody with Alexa Fluor 555 or 488 secondary antibodies (Cell Signaling Technology, Inc., Danvers, MA, USA) before observing under confocal microscopy (Leica, TCS-SP8, Leica).

### Measurement of the IFNγ concentration in the culture medium or the brain of mice

The IFNγ levels in the media of both control and pharmacologically treated cells or the brain of mice were determined using IFNγ enzyme immunoassay kits following the manufacturer’s instructions. The total protein used for ELISA was used as a loading control, and the results are expressed as pg of IFNγ per mg of total protein.

### Transfection

Cells were transfected with 100 nM of an ERK1/2- or p65-specific siRNA oligonucleotide. In control experiments, the cells were transfected with 100 nM scrambled siRNA. The transfected cells were allowed to recover for at least 12 h in growth medium and then incubated overnight in serum-free medium before extraction.

### Animal committee

All animals were handled according to the care and use of medical laboratory animals (Ministry of Health, Peoples Republic of China, 1998) and all experimental protocols were approved by the Laboratory Ethics Committees of College of Life and Health Sciences of Northeastern University.

### Human brain samples

Human brain samples were obtained from New York Brain Bank, serial numbers P535-00 (normal), TT4263 (early stage of AD, the patient is 73-years-old man who was diagnosed as a mild AD patient), T4308 (middle stage of AD, the patient is 86-years-old man who was diagnosed as moderate AD patient), T4339 and T4304 (late stage of AD, the patients are 88-years-old woman and 84 years-old woman who were diagnosed as severe and end stage of AD patients).

### Statistical analysis

All data are represented as the mean ± S.E. of at least three independent experiments. The statistical significance of the differences between the means was determined either using Student’s *t*-test^10^,^25^.

## Results

### IFNγ is markedly upregulated in APP/PS1 transgenic mouse brain

**Figure 1:**
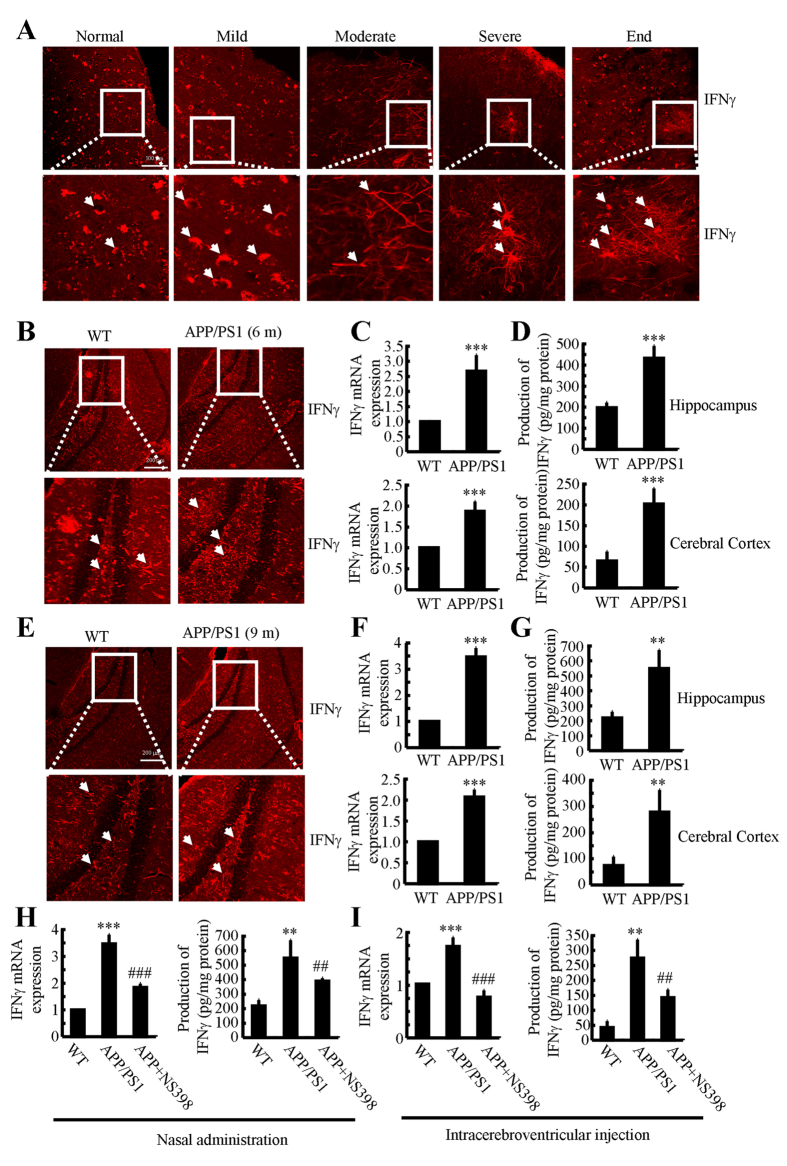
NS398 treatment decreases the induction of IFNγ in APP/PS1 mice. (**A**) The tissue blocks of human brains at different stages of AD were collected by the New York Brain Bank at Columbia University. Free-floating slices (40 μm) were prepared by cryostat. (**B–G**) The brains of WT or APP/PS1 transgenic mice at 6 or 9 months of age were collected following anesthesia and perfusion. In select experiments, the APP/PS1 transgenic mice at the age of 3 month received NS398 (50 μg/kg/d) intranasally for 6 months before brain harvesting (**H**). In separate experiments, APP/PS1 mice were injected (i.c.v) with NS398 (2 μg/5 μl) for 24 h (**I**). The immunoreactivity of IFNγ was determined by immunohistochemistry using an anti-IFNγ antibody (**A,B,E**). The arrows demonstrated the positive staining of IFNγ. IFNγ protein and mRNA levels were determined by IFNγ enzyme immunoassay kits and qRT-PCR, respectively (**C–I**). Total amounts of protein and GAPDH served as an internal control. The data represent the means ± S.E. of atleast three independent experiments. *p < *0.05*; **p < *0.01* and ***p < *0.001* with respect to WT control. ^#^*p* < *0.05*; ^##^*p* < *0.01* and ^###^*p* < *0.001* compared to APP/PS1 alone.

Due to previous studies suggesting that IFNγ plays a critical role in the pathogenesis of AD^21^, we evaluated the expression levels of IFNγ in AD patients and APP/PS1 transgenic mice at 6 or 9 months of age. As shown in Fig. 1A, IFNγ immunostaining was progressively induced during the course of AD development. Interestingly, the morphology analysis demonstrated that positive staining of IFNγ translocated from neurons to astrocytes. In line with these observations in AD patients, IFNγ immunostaining was highly enhanced in the cerebral cortex and dentate gyrus (DG) region of the hippocampus of APP/PS1 mice at 6 months of age when compared to WT C57BL/6 mice (Fig. 1B). These data reveal that IFNγ is upregulated with the development/progression of AD. To further confirm this finding, we examined the mRNA and protein levels of IFNγ in these APP/PS1 Tg mice. In agreement with the immunostaining data, our results demonstrated the upregulation of IFNγ mRNA and protein levels in the cerebral cortex and DG region of the hippocampus (Fig. 1C,D). In addition, we found that IFNγ was also stimulated in APP/PS1 mice at 9 months of age (Fig. 1E). Similarly, mRNA and protein levels of IFNγ were sustained above the basal levels (Fig. 1F,G). These observations indicate the possible role of Aβ aggregation in IFNγ stimulation.

### NS398 treatment attenuates the expression of IFNγ in APP/PS1 transgenic mice

Because COX-2 expression was elevated at the early stage of AD and was associated with Aβ deposition^34^, we studied whether COX-2 inhibition by NS398 downregulates the expression of IFNγ. We intranasally administered NS398 (50 μg/kg/d) to WT or APP/PS1 mice for 6 months prior to sacrifice. The results demonstrated that NS398 administration decreased the mRNA and protein expression of IFNγ (Fig. 1H). To further validate the above results, we injected (i.c.v) APP/PS1 mice at 6 months of age with NS398 (2 μg/5 μl). After 24 h, the brains of mice were collected and the expression of IFNγ was determined. The mRNA and protein expression of IFNγ was highly induced in the APP/PS1 mice, which was blocked by NS398 injection (Fig. 1I). These observations clearly indicate that COX-2 elevation stimulated the expression of IFNγ in APP/PS1 transgenic mice.

### PGE_2_ upregulates the expression of IFNγ, whereas PGI_2_ downregulates the expression of IFNγ

**Figure 2:**
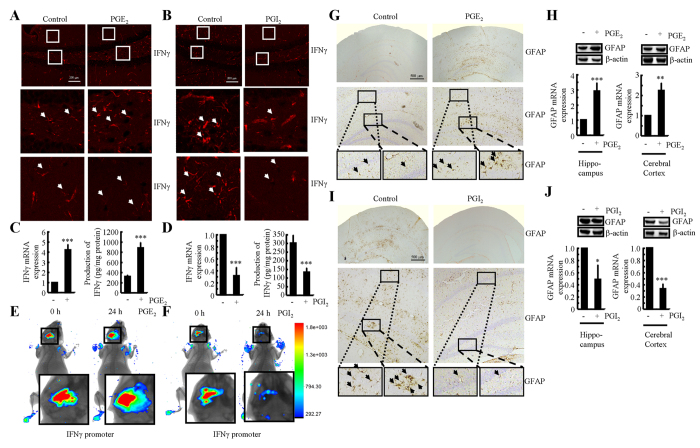
Antagonistic effects of PGE_2_ and PGI_2_ on regulating the expression of IFNγ in WT or APP/PS1 transgenic mice. The WT or APP/PS1 C57BL/6 mice at the age of 6 months were injected (i.c.v.) with PGE_2_ (2 μg/5 μl) or PGI_2_ (2 μg/5 μl). The brains were then collected and sectioned after 24 h (**A,B,G,I**). In select experiments, one side of the cerebral ventricle was injected with PGE_2_ (2 μg/5 μl) or PGI_2_ (2 μg/5 μl), and the other side of the cerebral ventricle was injected (i.c.v.) with D1A cells, which was pre-transfected with the IFNγ promoter (**E,F**). The immunoreactivity of IFNγ was determined by immunofluorescence staining using an anti-IFNγ antibody (**A,B**). Luciferase activities from the different groups of mice were measured using live animal imaging system (**E,F**). The activities of astrocytes were determined by immunohistochemistry with anti-GFAP (**G,I**). mRNA and protein levels of IFNγ and GFAP were determined by qRT-PCR, western blot and IFNγ enzyme immunoassay kits, respectively (**C,D,H,J**). Total amounts of GAPDH, β-actin and protein served as an internal control. The data represent the means ± S.E. of atleast three independent experiments. *p < *0.05*; **p < *0.01* and ***p < *0.001* with respect to PBS (−) or vehicle-treated controls.

Because NS398 treatment markedly decreased the expression of IFNγ in APP/PS1 mice at 6 months of age (Fig. 1H,I), we sought to determine the roles of COX-2 metabolic products, including PGE_2_ and PGI_2_, in regulating the expression of IFNγ following intracerebroventricular injection. It is evident that PGE_2_ (2 μg/5 μl) injection (i.c.v) stimulated the expression of IFNγ in the dentate gyrus (DG) region of hippocampus of mice (Fig. 2A). The mRNA and protein levels of IFNγ were detected using qRT-PCR and ELISA. The results showed that PGE_2_ injection (i.c.v) increased the expression of IFNγ in the cerebral cortex of WT mice (Fig. 2C). To further verify the key role of PGE_2_ in upregulating the expression of IFNγ *in vivo*, we combined i.c.v injection with live animal imaging. As described in Fig. 2E, D1A cells that were transfected with the IFNγ promoter constructs were pre-seeded in the left lateral ventricle of WT mice at 6 months of age, whereas PGE_2_ (2 μg/5 μl) was injected into the right ventricle of the same mice. After 24 h, luciferin was injected (i.c.v) into the side of cerebral ventricles of APP/PS1 Tg mice, which was pre-seeded with D1A cells before live animal imaging. The results showed that PGE_2_ increased the luciferase activity of the IFNγ promoter (Fig. 2E). To understand if the increased production of IFNγ was a result of microglia or astrocyte activation, we determined the activity of astrocytes following i.c.v injection of PGE_2_. The results demonstrated that astrocytes were markedly stimulated by PGE_2_ injection (Fig. 2G,H).

To further understand the roles of COX-2 metabolic products in IFNγ regulation, we similarly injected (i.c.v) PGI_2_ into the ventricles of 6-months-old APP/PS1 mice. In contrast to PGE_2_, PGI_2_ injection (i.c.v) decreased the positive staining of IFNγ in the cerebral cortex of APP/PS1 transgenic mice at 6 months of age (Fig. 2B). mRNA and protein levels of IFNγ were assessed using qRT-PCR and western blots. Similar results were obtained as in IHC assays (Fig. 2D). Additionally, PGI_2_ treatment actively alters the transcriptional activity of the IFNγ promoter and synthesis in live animals, as observed by live animal imaging (Fig. 2F). We then sought to understand the role of PGI_2_ in regulating the expression of IFNγ through the activity of astrocytes by quantifying their activity following injecting (i.c.v) with PGI_2_. As expected, the activity of astrocytes was suppressed by PGI_2_ injection (i.c.v) (Fig. 2I). In addition, PGI_2_ treatment suppressed the expression of GFAP in cerebral cortex and hippocampus (Fig. 2J). These observations not only demonstrated the opposing roles of PGE_2_ and PGI_2_ in regulating the expression of IFNγ, but also indicated the possible roles of astrocytes in expressing IFNγ.

### NF-κB nuclear translocation plays an important role in mediating the effects of PGE_2_ and PGI_2_ in regulating the expression of IFNγ in astrocytes

**Figure 3:**
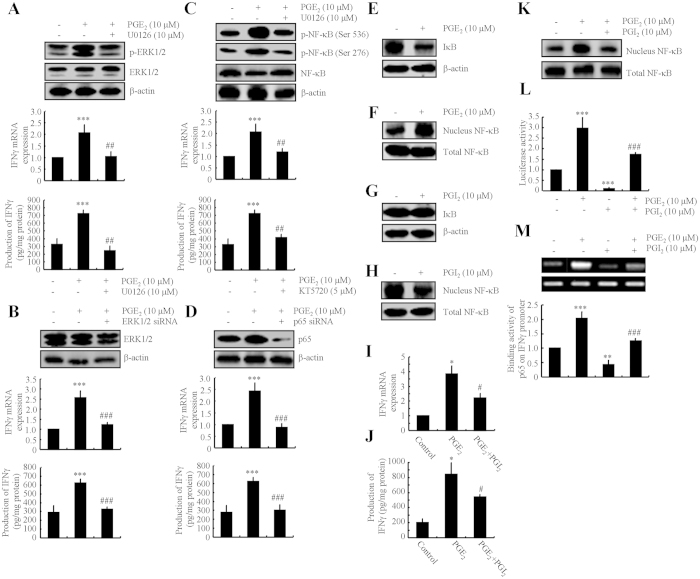
Critical roles of NF-κB activity in regulating IFNγ expression by PGE_2_- and PGI_2_-stimulated D1A cells. Mouse astrocyte D1A cells were treated with PGE_2_ (10 μM) in the absence or presence of the ERK1/2 inhibitor U0126 (10 μM) (**A,C** upper panel), KT5720 (5 μM) (**C** lower panel) for 24 h before extracting protein or mRNA (**A,C,E,F**). In select experiments, the cells were transfected with ERK1/2 or p65 siRNA before incubation with PGE_2_ (10 μM) for 24 h (**B,D**). In separate experiments, cells were treated with PGI_2_ (10 μM) for 24 h (**G,H)**. In distinct experiments, the cells were treated with PGE_2_ (10 μM) in the absence or presence of PGI_2_ (10 μM) for 24 h (**I–M**). Total ERK1/2 (**A,B**), phosphorylated ERK1/2 levels (**A**), total NF-κB **(C,D**), phosphorylated NF-κB (**C**) and total IκB (**E,G**) were detected by immunoblotting using specific antibodies. Equal lane loading was demonstrated by the similar intensities of total β-actin. The nuclear and total NF-κB levels were determined by western blots (**F,H,K**). IFNγ protein and mRNA levels were determined by IFNγ enzyme immunoassay kits and qRT-PCR, respectively (**A–D,I,J**). Total amounts of protein and GAPDH served as an internal control. The luciferase activity of the IFNγ promoter was determined by dual luciferase reporter assay kits (**L**). The binding activity of NF-κB to the promoter of IFNγ was determined by ChIP assay (**M**). The data represent the means ± S.E. of atleast three independent experiments. *p < *0.05*; **p < *0.01* and ***p < *0.001* with respect to the vehicle-treated or vector-transfected control. ^#^*p* < *0.05*; ^*##*^*p* < *0.01* and ^*###*^*p* < *0.001* compared to PGE_2_-treated alone.

As PGE_2_ and PGI_2_ demonstrated antagonistic effects on regulating the expression of IFNγ, we next determined the mechanism of IFNγ regulation by PGE_2_ and PGI_2_. Using D1A cell culture, we found that PGE_2_ treatment induced the phosphorylation of ERK1/2 without altering the total protein levels of ERK1/2 in D1A (Fig. 3A). To further elucidate the potential role of ERK1/2 in regulating the expression of IFNγ, we treated D1A cells with the pharmacological ERK1/2 inhibitor U0126 (10 μM) in the absence or presence of PGE_2_ (10 μM). Incubation of D1A cells with U0126 (10 μM) not only suppressed the PGE_2_-induced phosphorylation of ERK1/2 but also reversed the PGE_2_-stimulated IFNγ synthesis (Fig. 3A). To eliminate any potential non-specific effects of the pharmacological ERK1/2 inhibitor U0126, we conducted experiments with D1A cells that were transfected with an siRNA oligonucleotide sequence that was specific for ERK1/2. ERK1/2 knockdown and scramble control cells were treated with PGE_2_ (10 μM) or vehicle control for 48 h. ERK1/2 knockdown markedly reversed the stimulatory effects of PGE_2_ on the mRNA and protein expression of IFNγ in D1A cells (Fig. 3B).

To identify the mechanism of the transcriptional upregulation of IFNγ by PGE_2_, we determined the possible involvement of transcriptional factors in this process. Due to our previous observations^10^, we found that PGE_2_ treatment stimulates the phosphorylation of NF-κB at both Ser 536 and Ser 276 sites in D1A cells (Fig. 3C). The activation of NF-κB was blocked by U0126 treatment (Fig. 3C), which indicates the potential contribution of NF-κB in regulating IFNγ synthesis. To decipher the role of NF-κB in mediating IFNγ synthesis, we next treated D1A cells with the PKA inhibitor KT5720 (5 μM) in the absence or presence of PGE_2_ (10 μM). The results demonstrated that KT5720 treatment reversed the effects of PGE_2_-induced expression of IFNγ via suppressing the phosphorylation of NF-κB at the sites of Ser 536 and Ser 276 in D1A cells (Fig. 3C). The reason for using KT5720 to inhibit NF-κB is because NF-κB located downstream of PKA to exert biological function^35^,^36^. To eliminate any non-specific effects of KT5720 on the activity of NF-κB, we conducted experiments using cells that were transfected with an siRNA oligonucleotide that was specific for the NF-κB p65 subunit. The efficacy of the p65 knockdown was assessed through quantifying p65 protein levels in D1A cells (Fig. 3D upper panel). p65 knockdown reversed the stimulatory effects of PGE_2_ on the mRNA and protein expression of IFNγ in D1A cells (Fig. 3D lower panel). In particular, we found that PGE_2_ increased NF-κB translocation to the nucleus by decreasing the amount of IκB in D1A cells (Fig. 3E,F). In contrast, PGI_2_ decreased nuclear translocation of NF-κB without affecting the total amount of IκB in D1A cells (Fig. 3G,H). These data further support the notion that PGE_2_ and PGI_2_ have antagonistic effects on the regulation of IFNγ expression in a NF-κB-dependent manner.

### PGI_2_ attenuates the effects of PGE_2_ on stimulating the expression of IFNγ

In an effort to validate this hypothesis, we treated D1A cells with PGE_2_ in the absence or presence of PGI_2_. The results showed that PGI_2_ attenuated the effects of PGE_2_ on stimulating the expression of IFNγ (Fig. 3I). Although PGI_2_ reduced the expression of IFNγ in PGE_2_-injected mice, the level of IFNγ was still above the basal level (Fig. 3I). This observation was then confirmed using ELISA (Fig. 3J). As PGE_2_ and PGI_2_ demonstrated opposing effects on the phosphorylation and nuclear translocation of NF-κB (Fig. 3F, H), we sought to determine whether NF-κB transcriptionally mediated the effects of PGE_2_ and PGI_2_ on regulating the expression of IFNγ in D1A cells. We found that PGI_2_ attenuated the stimulatory effects of PGE_2_ on NF-κB nuclear translocation (Fig. 3K), which is consistent with the level of mRNA transcripts and protein synthesis of IFNγ (Fig. 3I, J). We also found that PGE_2_ upregulated the IFNγ promoter activity, whereas PGI_2_ downregulated the promoter activity of IFNγ (Fig. 3L). As expected, PGI_2_ attenuated the effects of PGE_2_ on stimulating the promoter activity of IFNγ (Fig. 3L). These data were further confirmed using chromatin immunoprecipitation assays (Fig. 3M).

### Aβ_1–42_ is involved in mediating PGE_2_- and PGI_2_-regulated IFNγ expression *via* an NF-κB-dependent mechanism

**Figure 4:**
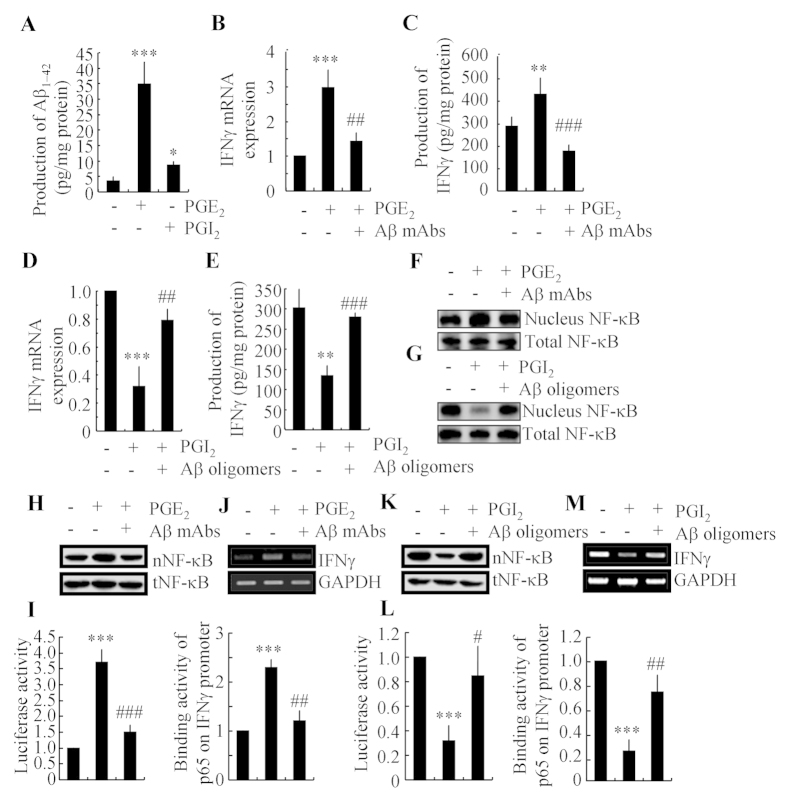
Aβ_1–42_ mediated the antagonistic effects of PGE_2_ and PGI_2_ on regulating the expression of IFNγ. D1A cells were treated with PGE_2_ (10 μM) or PGI_2_ (10 μM) for 48 h (**A**). In select experiments, PGE_2_ (2 μg/5 μl) or PGI_2_ (2 μg/5 μl) was injected (i.c.v.) into the ventricles of WT C57BL/6 or APP/PS1 mice in the absence or presence of Aβ antibody (1 μg/5 μl) or Aβ_1–42_ oligomers (1 μg/5 μl) for 24 h (**B**–**G**). In separate experiments, D1A cells were treated with PGE_2_ (10 μM) in the absence or presence of Aβ antibody (1 μg/ml) for 24 h (**H–J**). In distinct experiments, D1A cells were treated with PGI_2_ (10 μM) in the absence or presence of Aβ_1–42_ oligomers (1 μM) (**K**–**M**). The production of Aβ_1–42_ was determined by Aβ_1–42_ ELISA kits (**A**). Total amount of protein served as internal control. IFNγ protein and mRNA levels were determined by IFNγ enzyme immunoassay kits and qRT-PCR, respectively (**B–E**). Total amounts of protein and GAPDH served as an internal control. The nuclear and total NF-κB levels were determined by western blots (**F–K**). The luciferase activity of the IFNγ promoter was determined by dual luciferase reporter assay kits (**I,L**). The binding activity of NF-κB to the promoter of IFNγ was determined by ChIP assay (**J,M**). The data represent the means ± S.E. of atleast three independent experiments. *p < *0.05*; **p < *0.01* and ***p < *0.001* with respect to the vehicle-treated control. ^#^*p* < *0.05*; ^*##*^*p* < *0.01* and ^*###*^*p* < *0.001* compared to PGE_2_-treated alone.

As Aβ_1–42_ has an essential role in neuroinflammation^2^,^3^, we sought to determine the involvement of Aβ_1–42_ in mediating the effects of PGE_2_ and PGI_2_ on regulating the expression of IFNγ. Interestingly, we found that both PGE_2_ and PGI_2_ have the ability to stimulate the production of Aβ_1–42_ (Fig. 4A). However, PGI_2_ displayed a relatively weak ability to stimulate the production of Aβ_1–42_ when compared to PGE_2_. To further understand the role of Aβ_1–42_ in IFNγ regulation, we injected (i.c.v) PGE_2_ (2 μg/5 μl) into the ventricles of WT mice in the absence or presence of Aβ_1–42_ antibody (1 μg/5 μl). The results demonstrated that the Aβ_1–42_ antibody thoroughly diminished the stimulatory effects of PGE_2_ on IFNγ expression (Fig. 4B,C). Because PGI_2_ also increased the production of Aβ_1–42_, we then treated the APP/PS1 mice at 6 months of age with PGI_2_ (2 μg/5 μl) in the absence or presence of Aβ oligomers (1 μg/5 μl). The results showed that Aβ oligomers restore the decreasing expression of IFNγ in PGI_2_ injected (i.c.v) mice (Fig. 4D,E). As PGI_2_ stimulates the production of Aβ_1–42_, which is responsible for IFNγ synthesis, we sought to understand how elevated PGI_2_ levels depressed the expression of IFNγ while not stimulating its production. In view of our data showing the opposite effects of PGE_2_ and PGI_2_ on NF-κB nucleus translocation, we further determined NF-κB mobility in different groups of mice. The results revealed that Aβ antibody attenuated the PGE_2_-induced NF-κB nucleus translocation, whereas Aβ oligomers restored the suppressive effects of PGI_2_ on NF-κB nucleus translocation (Fig. 4F,G). To further confirm these *in vivo* observations, we treated the D1A cells with PGE_2_ in the absence or presence of Aβ_1–42_ antibody. The results demonstrated that Aβ_1–42_ antibody blocked the effects of PGE_2_ on stimulating the NF-κB nuclear translocation (Fig. 4H). However, Aβ_1–42_ oligomers administration increased the NF-κB nuclear translocation in PGI_2_-treated D1A cells (Fig. 4K). These data were further confirmed using promoter assay and chromatin immunoprecipitation assays (Fig. 4I–M). The results clearly demonstrated that PGI_2_ and Aβ have antagonistic effects on NF-κB transcriptional activation. Therefore, it is possible that the production of Aβ_1–42_ by PGI_2_ might not be sufficient to reverse the effects of PGI_2_ on inhibiting the NF-κB nuclear translocation and the expression of IFNγ. PGI_2_ does not always regulate IFNγ expression *via* Aβ_1–42_.

### Aβ_1–42_ oligomers stimulate the expression of IFNγ in APP/PS1 mouse brain

**Figure 5:**
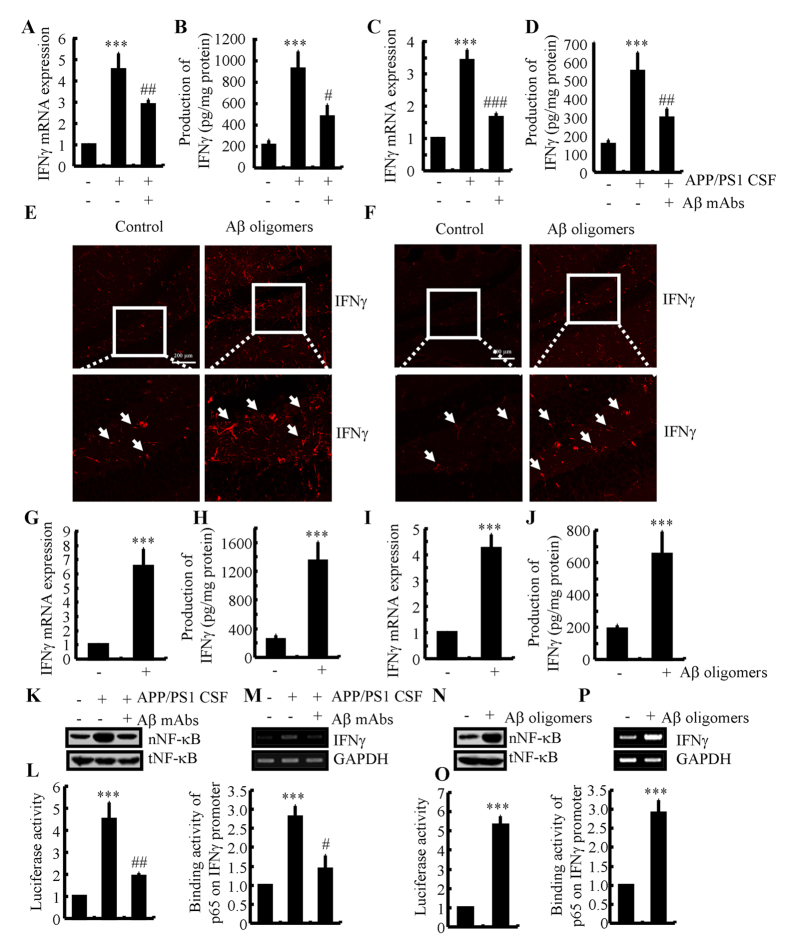
IFNγ upregulation at the early stage of AD was caused by Aβ oligomers. Cerebrospinal fluid (CSF) of APP/PS1 mice at 6 months of age was collected and then injected (i.c.v.) into wild type C57BL/6 mice in the absence or presence of Aβ antibody (1 μg/5 μl) for two weeks before sacrifice (**A,B**). In select experiments, D1A cells were treated with CSF of APP/PS1 mice at 6 months of age (1:1000 dilution) in the absence or presence of Aβ antibody (1 μg/ml) for 24 h (**C,D,K–M**). In separate experiments, the wild type C57BL/6 mice at the age of 6 months were injected (i.c.v) with Aβ oligomers (2 μg/5 μl) for 24 h (**E,G,H**). In distinct experiments, the slices of 6-month-old WT mice or D1A cells were cultured in Aβ_1–42 _oligomers (**F,I,J,N–P**). The immunoactivity of IFNγ was determined by an immunofluorescence assay (**E,F**). IFNγ protein and mRNA levels were determined by IFNγ enzyme immunoassay kits and qRT-PCR, respectively (**A–D,G–J**). Total amounts of protein and GAPDH served as an internal control. The nuclear and total NF-κB levels were determined by western blots (**K,N**). The luciferase activity of the IFNγ promoter was determined by dual luciferase reporter assay kits (**L,O**). The binding activity of NF-κB to the promoter of IFNγ was determined by ChIP assay (**M,P**). The data represent the means ± S.E. of atleast three independent experiments. *p < *0.05*; **p < *0.01* and ***p < *0.001* with respect to vehicle-treated controls. ^#^*p* < *0.05*; ^*##*^*p* < *0.01* and ^*###*^*p* < *0.001* compared to APP/PS1 CSF-treated alone.

Since Aβ involved in the roles of PGE_2_ and PGI_2_ in regulating the expression of IFNγ, we continued to determine the effects of different aggregated forms of Aβ on the expression of IFNγ in mice. As a first step, we determined the presence of aggregated forms of Aβ in CSF with thioflavin T staining. The results demonstrated that Aβ oligomers exist in the CSF of 6-months-old APP/PS1 mice (data not shown). This observation indicated that Aβ oligomers in CSF might be critical for IFNγ induction. To further validate this hypothesis, we injected CSF of the APP/PS1 at 6 months of age into WT mice in the absence or presence of Aβ antibody (1 μg/5 μl). After two weeks, the mice were sacrificed and determined the expression of IFNγ. Our data revealed that APP/PS1 CSF injection (i.c.v) elevated the expression of IFNγ, which was then blocked by the Aβ antibody (Fig. 5A,B). Similar results were also obtained in the APP/PS1 CSF-treated D1A cells (Fig. 5C,D). This observation indicates a critical role for the Aβ oligomers in CSF of APP/PS1 mice in the upregulation of IFNγ expression. To more clearly understand this mechanism, we injected Aβ oligomers (i.c.v) into the ventricles of WT mice. The results demonstrated that IFNγ expression was upregulated (Fig. 5E,G,H). In addition, Aβ oligomers (1 μM) treatment increased the expression of IFNγ in cultured slices (Fig. 5F). In agreement with these *in vivo* observations, Aβ oligomer treatment also induced the expression of IFNγ in D1A cells (Fig. 5I,J). More interestingly, we further found that Aβ mAbs blocked the effects of APP/PS1 mice CSF on stimulating NF-κB nucleus translocation and transcriptional activity by promoter and ChIP assays in D1A cells (Fig. 5K–M). Moreover, Aβ oligomers were further identified as critical molecules for NF-κB nucleus translocation and transcriptional activity (Fig. 5N–P). Collectively, our data clearly revealed the critical roles of Aβ oligomers in CSF of APP/PS1 mice in upregulating the expression of IFNγ.

### Aβ_1–42_ aggregation in plaques is critical for upregulating the expression of IFNγ in APP/PS1 mice

**Figure 6:**
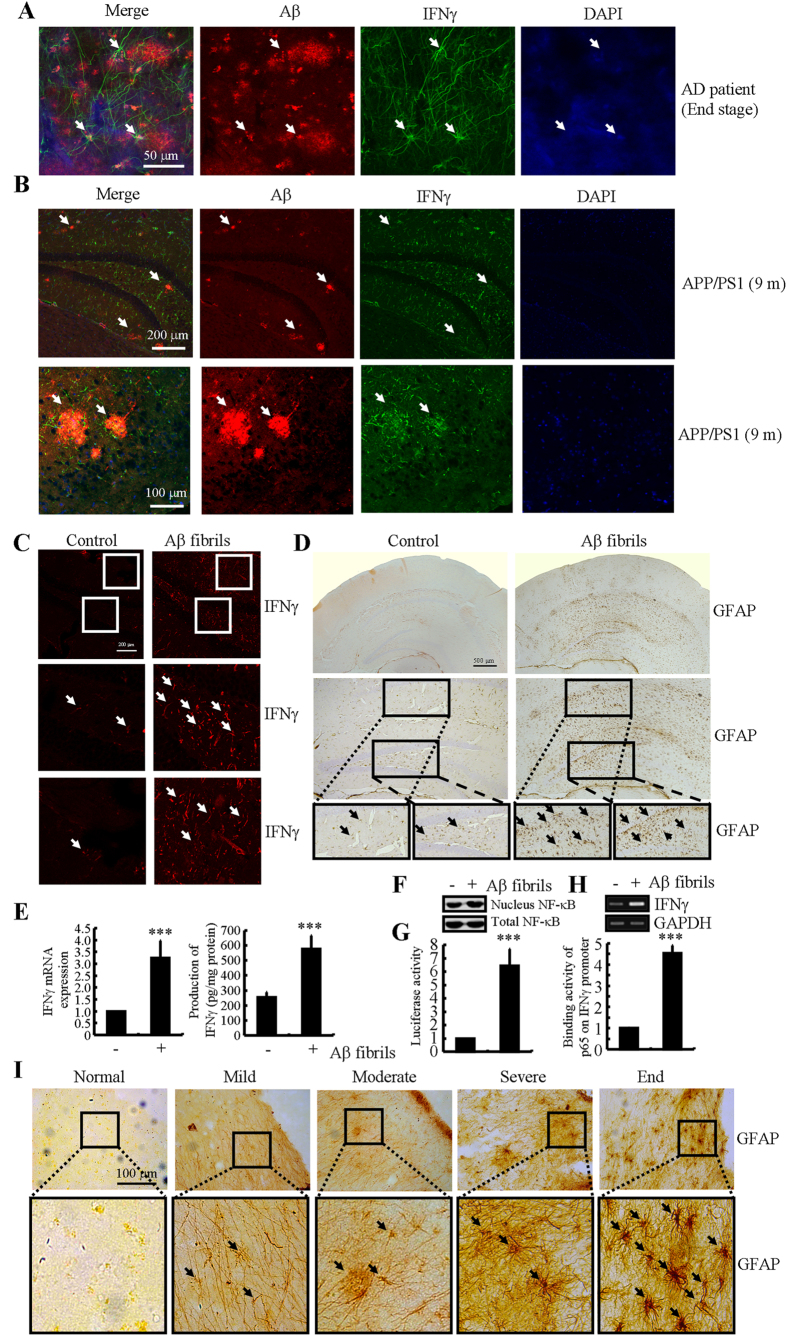
IFNγ upregulation at the late stage of AD was caused by advanced aggregated Aβ in APs. (**A,I**) The tissue blocks of human brains at different stages of AD were collected by the New York Brain Bank at Columbia University. Free-floating slices (40 μm) were prepared by cryostat. (**B**) The brains of WT or APP/PS1 transgenic mice at 9 months of age were collected after anesthesia and perfusion. In select experiments, brain slices of 6-month-old WT mice were cultured in the absence or presence of Aβ_1–42_ fibrils for 24 h (**C, D**). In separate experiments, D1A cells were incubated with Aβ fibers for 24 h (**E–H**). The slices of mouse brains were double-stained with Aβ (red) or IFNγ (green) antibodies before being observed under confocal microscopy (**A,B**). The immunoactivity of IFNγ was determined by an immunofluorescence assay (**C**). The activity of astrocytes was determined by staining with GFAP (**D,I**). IFNγ protein and mRNA levels were determined by IFNγ enzyme immunoassay kits and qRT-PCR, respectively (E). Total amounts of protein and GAPDH served as an internal control. The nuclear and total NF-κB levels were determined by western blots (**F**). The luciferase activity of the IFNγ promoter was determined by a dual luciferase reporter assay kits (**G**). The binding activity of NF-κB to the promoter of IFNγ was determined by a ChIP assay (**H**). The data represent the means ± S.E. of atleast three independent experiments. *p < *0.05*; **p < *0.01* and ***p < *0.001* with respect to vehicle-treated controls.

Because IFNγ was progressively upregulated during the course of AD development, we sought to understand the role of Aβ fibrils or APs in upregulating the expression of IFNγ. Therefore, we first found that IFNγ was stimulated around the APs either in AD patients or 9-months-old APP/PS1 transgenic mice (Fig. 6A,B). This observation clearly indicates that APs or advanced aggregates of Aβ_1–42_ have the ability to stimulate the expression of IFNγ by activating astrocytes. To further explore the role of the advanced aggregate form of Aβ_1–42_ in IFNγ regulation, we sliced fresh brain specimens from WT mice (400 μm) for culturing. The results demonstrated that IFNγ was activated by Aβ_1–42_ fibrils after 24 h of treatment (Fig. 6C). Similar results were obtained in D1A cells (Fig. 6E). In addition, the activities of astrocytes were stimulated by Aβ fibrils treatment (Fig. 6D). More importantly, the activities of astrocytes were progressively upregulated in AD patients (Fig. 6I). To further elucidate this mechanism, we conducted experiments to determine the effects of Aβ fibrils on NF-κB transcriptional activity. The results demonstrated that Aβ fibrils stimulate the activity of the IFNγ promoter by activating NF-κB in D1A cells (Fig. 6F-G). Therefore, our data revealed that not only Aβ_1–42_ oligomers but also Aβ_1–42_ fibrils have the ability to stimulate IFNγ expression by activating astrocytes, which produce high levels of IFNγ during the course of AD development.

### IFNγ overproduction accelerates the progression of AD development

**Figure 7:**
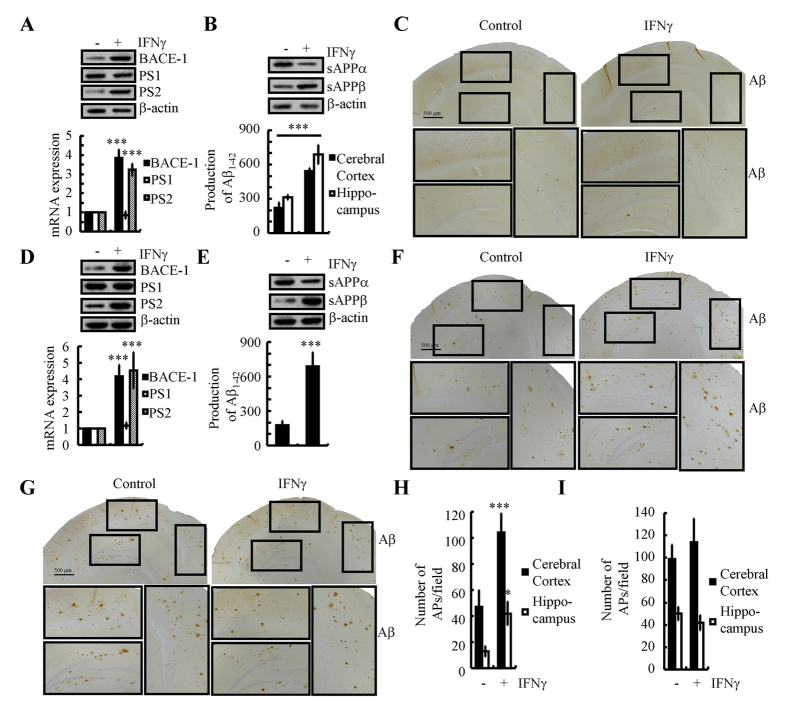
Nasal administration of IFNγ accelerates Aβ deposition in the brains of APP/PS1 mice by inducing the expression of BACE-1 and PS2. IFNγ (10 ng/20 μl/d) was nasally administered to 3-months-old WT mice for 7 days (**A–C**). In select experiments, n2a cells were treated with IFNγ (10 ng/ml) for 24 h before extracting total mRNA and protein (**D,E**). In separate experiments, 3-months-old APP/PS1 mice was nasally administered with IFNγ (10 ng/20 μl/d) for 3 or 6 months before determining the Aβ deposition in APs (**F–I**). The protein and mRNA expression of BACE-1, PS1 and PS2 were determined by western blot and qRT-PCR (**A,D**). Total amounts of β-actin and GAPDH served as an internal control. The production of sAPPα, sAPPβ and Aβ_1–42_ was determined by western blot and Aβ_1–42_ enzyme immunoassay kits (**B,E**). Total amounts of β-actin and protein served as an internal control. The immunoactivity of Aβ was determined by an immunohistochemistry assay (**C,F,G**). APs/field in cerebral cortex and hippocampus of APP/PS1 mice were analyzed by counting the number of APs in the images of immunohistochemistry assay (**H,I**). The data represent the means ± S.E. of atleast three independent experiments. *p < *0.05*; **p < *0.01* and ***p < *0.001* with respect to vehicle-treated controls.

As the mechanisms of IFNγ induction during the course of AD development in APP/PS1 mice had been elucidated, we are prompted to investigate the roles of IFNγ in Aβ deposition. To achieve brain drug delivery, human IFNγ was intranasal administered to the APP/PS1 mice. After 24 h, the brains were collected and sliced by cryostats. To determine if IFNγ achieve the brains of APP/PS1 mice, the slices were stained with antibody specific reactive with human IFNγ. The results demonstrated that IFNγ not only presented in the interstitial fluid but also on the neuronal cells (data not shown). The results demonstrated that intranasally administered IFNγ (10 ng/20 μl/d) for 7 days clearly increased the expression of BACE-1 and PS2, which resulted in accelerating the β-cleavage of APP and the production of Aβ_1–42_ (Fig. 7A–C). This *in vivo* observation was further verified in n2a cells (Fig. 7D,E). To further explore its roles in Aβ aggregation, we further treated APP/PS1 mice at the age of 3-months-old for 3 months or 6 months. The results demonstrated that Aβ deposition in APs is clearly elevated after 3-months-treatment, but not 6-months-treatment (Fig. 7F–I). Of note, we didn’t treat WT mice with IFNγ since the production of Aβ_1–42_ from WT mice might not have ability to aggregate or propagate. These observations clearly demonstrated that IFNγ overproduction accelerate the production and aggregation of Aβ_1–42_ in APs, which exacerbate the development of AD.

**Figure 8:**
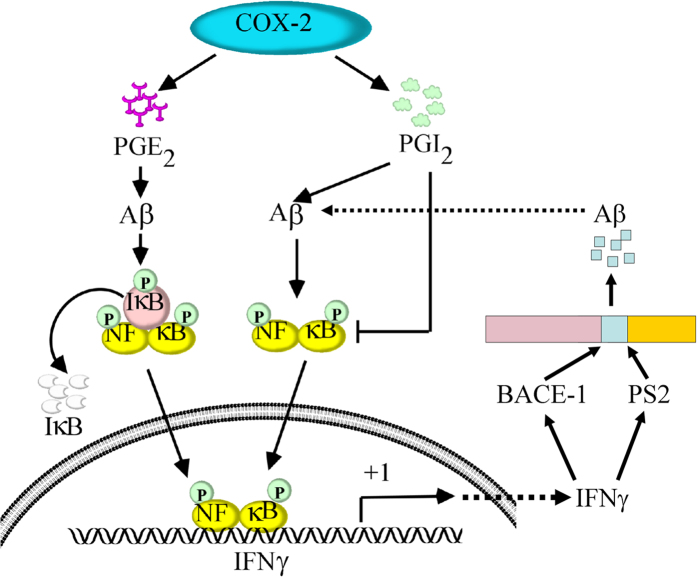
Signaling cascade of IFNγ upregulation during the course of AD development. COX-2 metabolic products including PGE_2_ and PGI_2_ displayed opposing effects on regulating the expression of IFNγ *in vitro* and *in vivo*. Specifically, PGE_2_ upregulated the expression of IFNγ via an Aβ-dependent NF-κB activating pathways. Although PGI_2_ can stimulate the expression of IFNγ via an Aβ-dependent NF-κB mechanism, PGI_2_ predominantly suppresses the expression of IFNγ via NF-κB-deactivating pathways, which is independent of Aβ_1–42_. Due to the role of PGE_2_ and PGI_2_ in inducing the production of Aβ_1–42_, we further found that Aβ_1–42_ oligomers stimulated the expression of IFNγ during the early stage of AD and that Aβ_1–42_ fibrils upregulated the expression of IFNγ at the late stage of AD. Highly expressed IFNγ accelerates the aggregation of Aβ_1–42_ in APs by inducing the expression of BACE-1 and PS2. These findings are instrumental for understanding the mechanisms of IFNγ upregulation in APP/PS1 transgenic mice and the roles of IFNγ in Aβ_1–42_ deposition in APs during the course of AD progression.

When considered together, our data revealed that PGE_2_ stimulates the synthesis of IFNγ via Aβ-dependent NF-κB activation pathways. Additionally, PGI_2_ attenuated the effects of PGE_2_ on stimulating the expression of IFNγ by decreasing the nuclear translocation of NF-κB. Although PGI_2_ has the ability to upregulate the production of Aβ_1–42_, the induced Aβ_1–42_ could not reverse the inhibitory effects of PGI_2_ on IFNγ expression. In line with these *in vitro* and *in vivo* observations, IFNγ was further found to be responsible for accelerating the production and deposition of Aβ_1–42_. More importantly, both Aβ_1–42_ oligomers and Aβ_1–42_ fibrils have the ability to stimulate the expression of IFNγ, which potentially aggravate the pathogenesis of AD by accelerating the Aβ deposition in APs (Fig. 8).

## Discussion

Prior work has revealed an early induction of COX-2 and of its metabolic products during the course of AD development^37^. Therefore, we studied the role of COX-2 and its metabolic products in AD. As a powerful inducer of inflammation, COX-2 has been shown to affect the expression of IFNγ *via* its metabolic products^13^. So, we investigated the role of PGE_2_ and PGI_2_ in regulating the expression of IFNγ during the course of AD development. Specifically, PGE_2_ stimulates the expression of IFNγ *via* Aβ_1–42_-dependent NF-κB activating pathways. In contrast, PGI_2_ attenuates the effects of PGE_2_ on inducing the expression of IFNγ in an NF-κB transactivating mechanism. Although Aβ_1–42_ reliably induces the expression of IFNγ by activating NF-κB, PGI_2_-induced Aβ_1–42_ might not be sufficient to reverse the inhibitory effects of PGI_2_. In agreement with these *in vitro* observations, we found that PGE_2_ and PGI_2_ antagonistically regulated the expression of IFNγ in an Aβ_1–42_-dependent manner. Moreover, both Aβ_1–42_ oligomers and Aβ fibrils have the ability to upregulate the expression of IFNγ, which results in constitutively high levels of IFNγ during the course of AD development.

IFNγ is tightly regulated under physiological conditions. Although the mechanisms of IFNγ upregulation and the role of IFNγ in AD are not fully understood, it has been demonstrated that IFNγ is present or significantly elevated in the AD brain^38^ and that IFNγ may be necessary for AD pathogenesis^18^,^19^,^20^,^21^,^22^,^39^. In line with these observations, we found that when compared with Aβ deposition at 6 months of age, IFNγ was highly induced in APP/PS1 mice at 6 months of age (and occurs earlier in the cerebral cortex). In agreement with our data, Abbas *et al.*^38^ reported that high levels of IFNγ production appeared early in the cerebral cortex (at 9 months) when compared to APs (generally at 11 months) in Tg2576 mice. Of note, this observation was supported by a series of investigations that demonstrated that IFNγ levels are increased in APP transgenic mouse brain^40^,^41^. Additionally, many IFNγ-responsive genes are upregulated in AD brain^38^,^42^,^43^. Unfortunately, this study was unable to determine how IFNγ was upregulated in the early stage of AD. For this reason, we extended the prior works to reveal the role of Aβ oligomers in IFNγ induction at the early stage of AD. At the late stage of AD, Aβ fibrils are responsible for IFNγ stimulation, which sustained constitutively high levels of IFNγ during the course of AD development.

However, we cannot conclude that IFNγ is temporarily stimulated at the early stage of AD. As expected, we found that APs have the ability to stimulate the expression of IFNγ by activating astrocytes in APP/PS1 transgenic mice at 9 months of age. In line with our observations, it is reported that increased IFNγ production occurs in the cerebral cortex of 17–19-month-old Tg2576 mice. Here, it was observed that active astrocytes surround the β-amyloid deposits^38^. According to this report, the highly aggregated form of Aβ_1–42_ might be critical for IFNγ elevation. Our results show that Aβ_1–42_ fibril treatment increased the expression of IFNγ expression by activating astrocytes. Yet, IFNγ is not only a passive molecule, as IFNγ has been suggested to regulate the pathogenesis of AD^18^. As a multiple immunoregulatory cytokine, IFNγ usually promotes the expression of other proinflammatory cytokines including TNF-α and IL-1, whose expression synergistically amplifies the effects of IFNγ on the production of Aβ_1–42_. In line with this hypothesis, Blasko *et al.*^39^ reported that costimulating human astrocytes with IFNγ, IL-1β and TNF-α increases the synthesis of Aβ_1–42_ and Aβ_1–40_. Therefore, the sensitive induction of Aβ_1–42_ following co-treatment of astrocytes with IFNγ and TNF-α is due to the upregulation of BACE-1^21^,^44^. In line with these observations, we further found that IFNγ administration has ability to enhance the Aβ_1–42_ production by increasing the expression of BACE-1 and PS2. Interestingly, our data further revealed that IFNγ accelerated the aggregation of Aβ_1–42_, but not affect the number of APs at the late stage of AD.

To keep the discussion focused, we will continue to elucidate the mechanisms of IFNγ upregulation in APP/PS1 transgenic mice. Due to the possible involvement of COX-2 metabolic products in Aβ deposition^45^,^46^,^47^, it is easier to speculate that COX-2 signaling might be critical for IFNγ upregulation via acceleration of Aβ deposition. In agreement with this hypothesis, prior work has shown that NSAID treatment decreases the production of Aβ_1–42_ in mice^48^. Specifically, celecoxib and rofecoxib treatment decreases the deposition of Aβ_1–42_ in AD patients and mouse models^49^,^50^. The ratio of Aβ_1–42_ and Aβ_1–40_ was also elevated in COX-2/APP/PS1 mice^34^. An *in vitro* assay revealed that PGH_2_ has the ability to induce the production of Aβ_1–42_^51^. In addition, PGE_2_ treatment increases the production of Aβ_1–42_ either in primary cultured mouse microglia^52^ or in C57BL/6 mice^53^. Given the critical roles of PGE_2_ in Aβ_1–42_ production and deposition, we predict a possible role for PGE_2_ in IFNγ upregulation. As a consequence, our results demonstrate that PGE_2_ treatment increases the expression of IFNγ in either astrocytes or in C57BL/6 mice. In agreement with these observations, PGE_2_ treatment increases the expression of IFNγ in primary cultured rat astrocytes^13^. Along these lines, Aβ_1–42_ deposition might be critical for the roles of PGE_2_ in upregulating the expression of IFNγ.

Interestingly, in contrast to PGE_2_, PGI_2_ shows suppressive effects on the expression of IFNγ. In agreement with these observations, Strassheim *et al.*^17^ reported that PGI_2_ inhibits interferon γ (IFNγ)-stimulated cytokine expression in human monocytes. Although we could not find other evidence that suggests that PGI_2_ has the ability to regulate the expression of IFNγ, it has been shown to inhibit neuroinflammation. For example, treatment with PGI_2_ analogs, including iloprost and treprostinil, suppressed TNF-α expression in human myeloid dendritic cells^9^. More closely, Wahlstrom *et al.*^15^ reported that the administration of the PGI_2_ analogue epoprostenol significantly decreased C-reactive protein (CRP) and generally decreased IL-6 levels in patients with severe traumatic brain injury compared to placebo. Schuh *et al.* 16 also reported that the early induction of PGI_2_ at the site of traumatic injury resulted in the aggregation of IL-1β-expressing macrophages as a critical reason for neuropathic pain.

Because PGE_2_ and PGI_2_ show antagonistic effects on the expression of IFNγ, it is possible that PGE_2_ and PGI_2_ have the ability to regulate the activity of astrocytes or microglia. To this end, we further found that PGE_2_ stimulates the activity of astrocytes by inducing the expression of GFAP. Although there is no direct evidence that supports our data, PGE_2_ treatment stimulates the activity of cultured astrocytes by elevating the levels of GFAP^54^. In contrast, PGI_2_ suppresses the activity of astrocytes by reducing the expression of GFAP^55^,^56^. The trends of astrocytes activity were similar to that of IFNγ expression. These observations not only indicated that IFNγ was produced from astrocytes, but also implied that the expression of IFNγ stimulates the activity of astrocytes. Additionally, Tsuda *et al.*^57^ reported that IFNγ signaling mediates spinal microglia activation, which is responsible for neuropathic pain. In contrast to microglia activation, IFNγ shows a modest induction of GFAP^58^. Given the important role of IFNγ in activating microglia and astrocytes, the receptors involved in IFNγ signaling are important. Hashioka *et al.*^59^ reported that almost all IFNγ-receptor-positive cells corresponded to GFAP-positive astrocytes, whereas none of the IFNγ-receptor cells corresponded to Iba1-positive microglia cells *in vivo*. In contrast to the *in vivo* results, almost all IFNγ-receptor cells were Iba1- and GFAP-positive in cultured microglia cells^59^.

Due to these observations, we next studied the involvement of NF-κB activity in regulating the expression of IFNγ. In line with the current study, our prior work has shown that PGE_2_ stimulates the expression of IL-1β by activating the NF-κB p65 subunit in glia^10^. In contrast, Raychaudhuri *et al.*^60^ reported that the PGI_2_ analogue treprostinil blocks NF-κB nuclear translocation in human alveolar macrophages. These observations are in agreement with our data, which suggests that PGE_2_ and PGI_2_ antagonistically regulate the activity of NF-κB. As PGE_2_ and PGI_2_ have the ability to induce the production of Aβ_1–42_, we demonstrated that Aβ_1–42_ stimulates NF-κB activity. Aβ_1–42_ has been previously reported to activate NF-κB activity in neuroblastoma SH-SY5Y cells^61^. Due to the important role of NF-κB in activating the IFNγ promoter^62^, we further found that NF-κB is important for the regulation of IFNγ expression in D1a cells.

In conclusion, this study provides new evidence for the antagonistic roles of PGE_2_ and PGI_2_ in regulating the expression of IFNγ *in vitro* and *in vivo*. Specifically, PGE_2_ upregulates the expression of IFNγ via an Aβ-dependent NF-κB activating pathway. In contrast, PGI_2_ attenuated the effects of PGE_2_ on stimulating the expression of IFNγ. As PGI_2_ displays only a modest induction of Aβ_1–42_, Aβ_1–42_ induction was insufficient to alleviate the cells from IFNγ inhibition by PGI_2_ in an NF-κB-dependent manner. These findings provide new insights into the mechanisms of IFNγ regulation in the bran during the course of AD development.

## Additional Information

**How to cite this article**: Wang, P. *et al.* Prostaglandin I_2_ Attenuates Prostaglandin E_2_-Stimulated Expression of Interferon γ in a β-Amyloid Protein- and NF-κB-Dependent Mechanism. *Sci. Rep.* **6**, 20879; doi: 10.1038/srep20879 (2016).
