# Addendum: Prostaglandin I_2_ Attenuates Prostaglandin E_2_-Stimulated Expression of Interferon γ in a β-Amyloid Protein- and NF-κB-Dependent Mechanism

**DOI:** 10.1038/s41598-022-12462-4

**Published:** 2022-05-19

**Authors:** Pu Wang, Pei-Pei Guan, Xin Yu, Li-Chao Zhang, Ya-Nan Su, Zhan-You Wang

**Affiliations:** College of Life and Health Sciences, Northeastern University, Shenyang, 110819 People’s Republic of China

Addendum to: *Scientific Reports* https://doi.org/10.1038/srep20879, published online 12 February 2016

Concerns have been raised post-publication about the similarity between the WT and APP/PS1 images in Figure 1B. The Editors were unable to unequivocally confirm that the images are duplicated; at the same time, high resolution images are no longer available for inspection. However, the Authors were able to provide the data for eight other replicates from this study, which further supports the conclusions of the paper. This data is shown below as Figure 1.

**Figure 1:**
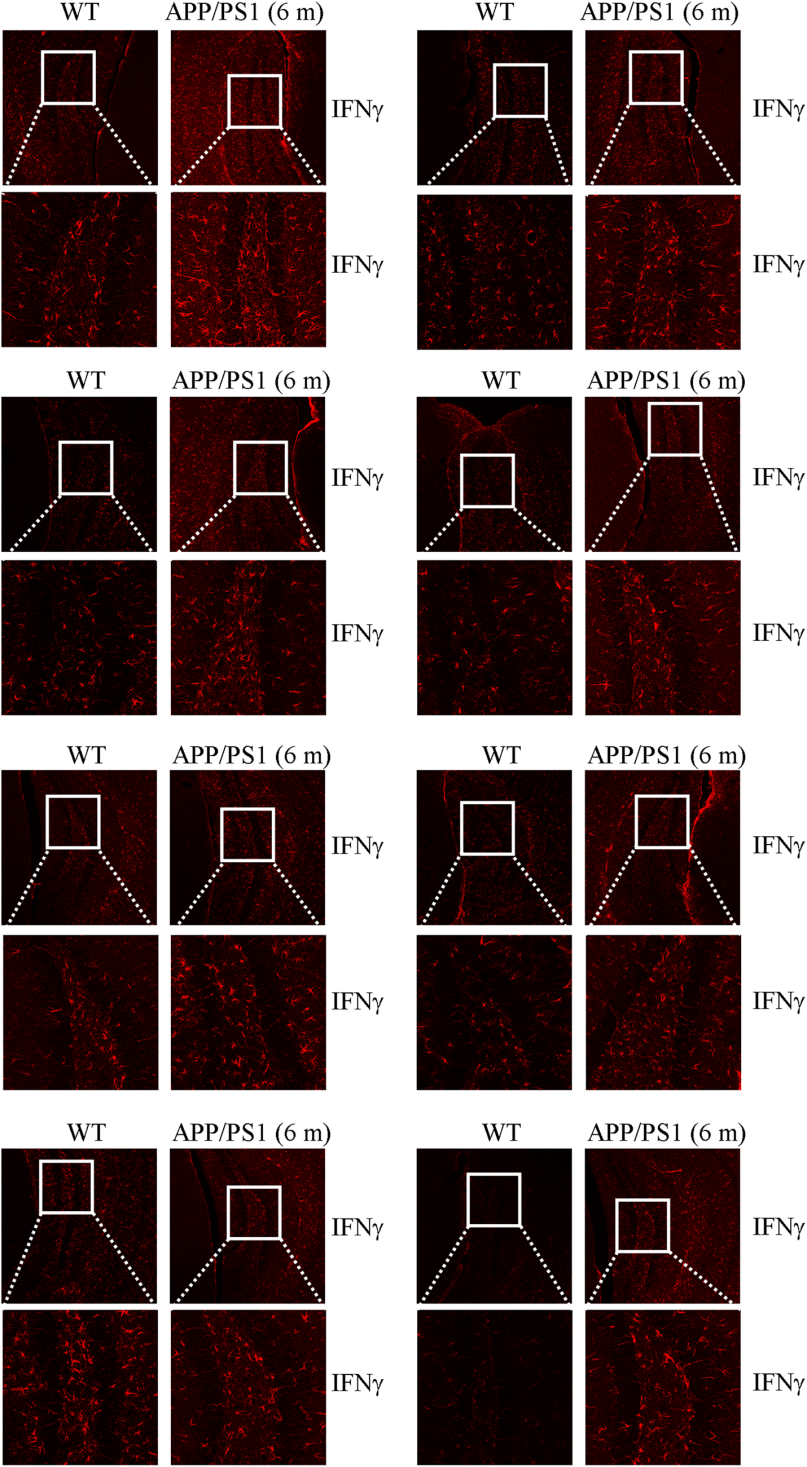
Additional replicates for the experiment depicted in Figure 1B in the Article.

